# Pattern separation and pattern completion: Behaviorally separable processes?

**DOI:** 10.3758/s13421-020-01072-y

**Published:** 2020-07-29

**Authors:** Chi T. Ngo, Sebastian Michelmann, Ingrid R. Olson, Nora S. Newcombe

**Affiliations:** 1 Department of Psychology, Temple University, Philadelphia, PA USA; 2 Center for Lifespan Psychology, Max Planck Institute for Human Development, Lentzeallee 94, 14195 Berlin, Germany; 3 Princeton Neuroscience Institute, Princeton University, Princeton, NJ USA

**Keywords:** Pattern separation, Pattern completion, Lure classification, Holistic recollection, Episodic memory

## Abstract

Episodic memory capacity requires several processes, including mnemonic discrimination of similar experiences, termed *pattern separation*, and holistic retrieval of multidimensional experiences given a cue, termed *pattern completion*. Both computations seem to rely on the hippocampus proper, but they also seem to be instantiated by distinct hippocampal subfields. Thus, we investigated whether individual differences in behavioral expressions of pattern separation and pattern completion were correlated after accounting for general mnemonic ability. Young adult participants learned events comprised of a scene-animal-object triad. In the pattern separation task, we estimated mnemonic discrimination using lure classification for events that contained a similar lure element. In the pattern completion task, we estimated holistic recollection using dependency in retrieval success for different associations from the same event. Although overall accuracies for the two tasks correlated as expected, specific measures of individual variation in holistic retrieval and mnemonic discrimination did not correlate, suggesting that these two processes involve distinguishable properties of episodic memory.

**Electronic supplementary material:**

The online version of this article (10.3758/s13421-020-01072-y) contains supplementary material, which is available to authorized users.

## Introduction

Episodic memory binds together the diverse co-occurring elements that make up the specific events of our lives, forming distinctive and complex events that can guide ongoing behavior. This capacity requires many processes. Past experiences can share overlapping content, hampering the retrieval of a specific event against the backdrop of interfering memories. Thus, remembering a past event with high specificity is optimized by pattern separation processes, whereby similar experiences are assigned to non-overlapping neural codes in the service of preserving each event’s distinctiveness. Further, episodic memory retrieval unites various aspects of an event including where you were, specific people you met, and the objects that were encountered as an integrated unit (Tulving, 2002). Pattern completion processes enables this network of relations to be retrieved holistically, such that one constituent of an event can elicit the retrieval of other elements from the same event. Computational models posit that the hippocampus instantiates two crucial computations of *pattern separation* and *pattern completion* to support mnemonic discrimination and holistic recollection. Critically, hippocampal subfields differentially participate in these processes (Montaldi & Mayes, 2010; Neunuebel & Knierim, 2014; O’Reilly & McClelland, 1994; Rolls, 2016).

### Mnemonic discrimination via pattern separation

Accurate episodic memory requires remembering details with high specificity, so that they can be mnemonically discriminated from other similar memories to circumvent catastrophic interference. Pattern separation aids mnemonic discrimination by reducing the degree of representational similarity among overlapping experiences. The granule cells in the dentate gyrus (DG) assign distinct representations to similar inputs via sparse coding, thereby amplifying small differences from the entorhinal cortex. The pattern-separated signals are then projected onto CA3 via the mossy fiber pathway. This mechanism helps minimize interference to support new learning (Marr, 1971; McClelland, McNaughton, & O’Reilly, 1995; Norman & O’Reilly, 2003).

In humans, one paradigm designed to index the behavioral outcome of pattern separation is the Mnemonic Similarity Task (MST). In this paradigm, participants first view a series of object images. At test, participants are shown targets (identical to the objects seen at encoding), lures (similar exemplars of the objects seen at encoding), and foils (new and dissimilar from those seen at encoding). The theoretically important part of this task is that it requires high-resolution mnemonic representations of studied objects in order to reject the highly-similar lure items. The ability to make this discrimination may relate to the DG/CA3 signals of the hippocampus, such that large responses were evoked by small changes in perceptual input (Lacy, Yassa, Stark, Muftuler, and Stark, 2011; Reagh & Yassa, 2014; reviewed in Yassa & Stark, 2011). Evidence from a study using ultra-high-resolution functional magnetic resonance imaging (fMRI) and multivariate pattern analysis further revealed that DG signals represent similar scenes in a less overlapping fashion compared to other hippocampal subfields and medial temporal regions (Berron et al., 2016). Causal evidence for a role of the DG in pattern separation comes from a case study of an individual with selective DG damage. This individual was impaired at making fine-grained target-lure discriminations (Baker et al., 2016), corroborating findings in rodents (Gilbert, Kesner, & Lee, 2001).

### Holistic recollection via pattern completion

Retrieving a complex and multimodal episode also necessitates pattern completion, which occurs when a partial cue reactivates a complete event representation (Marr, 1971; McClelland, McNaughton, & O’Reilly, 1995; Norman & O’Reilly, 2003). Pattern completion is thought to rely in part on hippocampal CA3 network, which receives pattern-separated inputs from the DG as well as direct inputs from the entorhinal cortex via the perforant pathway, bypassing the DG. The CA3 pyramidal cells project onto themselves via the recurrent collaterals. This mechanism is thought to associate elements of an event to a shared representation in CA3, thereby enabling the recapitulation of the conjunctive representation of an entire event from a partial cue (Guzowski, Knierim, & Moser, 2004; Rolls, 2016). An important property of pattern completion is that a whole memory can be initiated from any part of the memory (Rolls, 2016).

One paradigm to test pattern completion in humans relies on the conceptualization that pattern completion enables the recovery of the entire event based on a partial cue so that all elements within an event can be retrieved successfully (or not). For instance, if retrieving a place successfully reminds us of the person we met there, it would also likely evoke our memory of what objects we encountered in the same event. Findings from multiple studies have demonstrated that young adults indeed retrieve events in a holistic fashion (Bisby, Horner, Bush, & Burgess, 2018; Horner & Burgess, 2013, 2014; Ngo, Horner, Newcombe, & Olson, 2019). In the multi-element paradigm, participants first learned unique events, each comprised of a scene-person-object triad (e.g., kitchen-Obama-hammer). Later, participants performed a cued recognition task that included every possible cue-test element combination of each event (e.g., cue: kitchen, test: Obama), allowing for the examination of retrieval dependency between different associations of the same event. Evidence for significant retrieval dependency suggests that complex episodic memories may be retrieved as integrated units.

Findings on the neural bases of retrieval dependency point to the involvement of the hippocampus (Horner et al., 2015), and specifically the CA3 subfield (Grande et al., 2019). In a multi-element event task variant, participants learned scene-person-object triads (e.g., kitchen-Obama-hammer) in three separate trials of pairwise associations (e.g., kitchen-Obama; Obama-hammer; kitchen-hammer) along the encoding phase. After having seen the first two pairs of an event (e.g., kitchen-Obama, Obama-hammer), hippocampal activity during encoding of the final pair (e.g., kitchen-hammer) was associated with memory performance on other pairs of the same event. Furthermore, during cued recognition of pairwise associations (e.g., cue A; test B), neocortical activity corresponding to all event elements was reinstated, including the element that was incidental to a given trial (e.g., element C). Importantly, the extent of neocortical reinstatement of non-target elements correlated with hippocampal activity at retrieval, consistent with the presence of pattern completion (Horner et al., 2015; reviewed in Horner & Doeller, 2017). Crucially, this reinstatement signal is more robust within the CA3 compared to the dentate gyrus (Grande et al., 2019). Together, these findings support the idea that the retrieval of multiple elements of an event given the same cue is mutually contingent, and that this computation may, in part, rely on hippocampal pattern completion.

### Behavioral relation between pattern separation and completion

Pattern separation and completion are distinct computations that may support complementary aspects of episodic capacity: laying down high-resolution memory traces and holistic retrieval of an integrated unit, respectively. If the relevant computations support different episodic features of a memory, do their individual differences in these mnemonic capacities track one another? The answer to this question hinges upon the conceptual link between the behavioral consequences of pattern separation and completion, but thus far, the interpretation of this link has been mixed.

In some investigations, the behavioral proxies of pattern separation and pattern completion are thought to occupy two ends of a lure discrimination performance scale (Yassa & Stark, 2011). The inverse operational definitions for pattern separation and completion may have resulted in the problematic interpretation that an improvement in one process necessarily comes with the impairment in the other (for a discussion of this criticism, see Hunsaker & Kesner, 2013). For example, pattern separation failure measured by false alarms in lures trials on the MST is sometimes interpreted as a gravitation of the system towards pattern completion (e.g., Holden & Gilbert, 2012; Kirwan & Stark, 2007; Lacy et al., 2011; Reagh & Yassa, 2014; Yassa et al., 2010). Although pattern completion likely underlies false alarm response in lure trials (Wynn, Ryan, & Buchsbaum, 2020), such an approach in measuring pattern completion behaviorally may pertain to specific instances in which the memory judgment reflects a tradeoff between the two processes.

It is likely that in some cases, pattern separation and completion play complementary roles in supporting episodic memory accuracy by establishing distinctive memory traces and holistic retrieval of complex experiences. The measurement dependency between pattern separation and pattern completion in paradigms that use similar lures, by design, does not usually permit independent indices of these processes (but see Ally, Hussey, Ko, & Molitor, 2013). Thus, investigating the behavioral relation between pattern separation and pattern completion requires an operational definition of pattern completion that is independent of pattern separation failure hallmarks (e.g., Ally, Hussey, Ko, & Molitor, 2013; Rollins & Cloude, 2018; Vieweg, Stangl, Howard, & Wolbers, 2015).

### Current study

This study tested whether individual differences in holistic recollection were related to remembering the specific details of past events. In the same group of participants, we administered two tasks that shared the same encoding format. Participants learned a series of multi-element “events,” each consisting of a scene-animal-object triad. To operationalize pattern separation performance, we measured lure classification performance using a confusion matrix. We computed participants’ success in identifying the test events that included a lure element, i.e., lure classification. Importantly, a hallmark of pattern separation failure is confusing similar events with one another. Thus, we further corrected lure classification performance for the specific confusion patterns of misidentifying lure events as old events when measuring pattern separation. For pattern completion, we measured dependency – the degree to which the retrieval success of different associations from the same event is mutually contingent (all accurate or inaccurate).

We hypothesized that the behavioral expression of pattern separation through lure classification and pattern completion through holistic retrieval would not correlate with one another, as they were posited to rely on distinct neural computations. In addition, we tested whether general episodic memory performance that is nonspecific to pattern separation and completion would correlate between the two tasks. If this relation between gross accuracy of the two tasks exists, but the correlation between holistic retrieval and lure classification is not detected, this result would substantiate the idea that individual differences on pattern separation and pattern completion are unrelated. It is important to note, for naming heuristic purposes, pattern separation and pattern completion tasks here refer to the dependent variables of interest from each task, without the assumption that these processes are the only one at play in each task.

## Methods

### Participants

A total of 84 (42 female) undergraduate students (*M*_*a*ge_ = 20.07 years; *SD* = 2.06, range =18–27) from Temple University participated for partial course credit. All participants gave informed consent and reported to have normal or corrected-to-normal vision. This experiment was completed in accordance with and approved by the Institutional Review Board committee at Temple University. This sample size was determined based on a power analysis to ensure there was sufficient power (0.80) to detect significant Pearson correlations with a medium effect size (Cohen’s *d* =0.3) using G* power (see pre-registration).

### Memory task

#### Materials

##### Pattern separation task

We sampled 390 cartoon images of distinct scenes (e.g., living room), animals (e.g., panda), and object (e.g., comb) from Google image search engine. Every two images were pairs of perceptually similar exemplars (e.g., two perceptually similar living rooms). An independent group of 31 young adults rated the level of similarity of each exemplar pair in Qualtrics. We used the ranking results to assign scene-animal-object triad membership to minimize potential differences in difficulty levels, i.e., uneven levels of similarity, among different counterbalanced task versions across participants (see Online Supplementary Material 1.1). 360 images were used to create two sets of 60 events (sets A and B) such that event 1 in set A is made up of element exemplars of event 1 in set B. Four different task versions were created from the two sets of events to counterbalance the frequency with which each event would appear in the study list or in the test list as target or lure events, across participants. The other 30 stimuli were used to create ten foil events (events containing elements that only appear at the test phase), which were fixed across all counterbalanced task versions.

##### Pattern completion task

We sampled 72 cartoon images of distinct scenes, animals, and objects (24 per category) from Google image search engine to construct 24 scene-animal-object triads. The event assignment of the elements was randomized, with the exception that items with pre-experimental associations (e.g., books and library) were not assigned to the same event. The selected stimuli in this task did not overlap with those used in the pattern separation task.

#### Procedure

All participants were administered the pattern separation and pattern completion tasks in a counterbalanced order. Verbal instructions for both tasks were voice recorded.

##### Pattern separation task

**Fig. 1:**
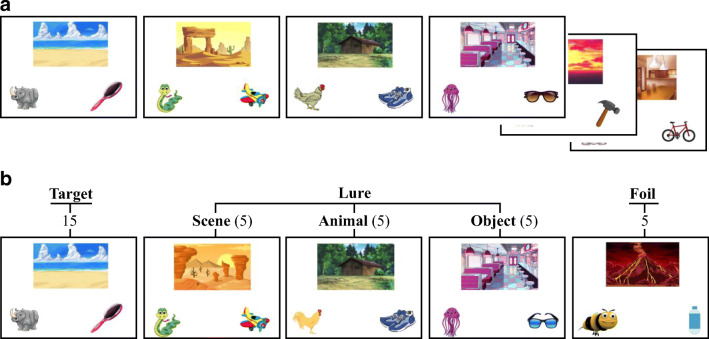
(**A**) A schematic depiction of the multi-element event task procedure for each of the two blocks. In each block, participants first learned 30 events, each comprised of a scene, an animal, and an object. (**B**) The test list consisted of 35 events including 15 target, five scene lures, five animal lures, five object lures, and five foil trials. Note that the stimuli between the two blocks were non-overlapping

The task procedure comprised of two encoding test blocks with non-overlapping stimuli between the two blocks. Each block consisted of 30 encoding and 35 test trials, which led to a total of 60 encoding and 70 test trials. At encoding, participants were instructed that they would see many different events, each consisting of a scene-animal-object triad, and that they should pay close attention to all of the different elements altogether in each event (see Figure 1A). A practice phase preceded the encoding phase in order to acquaint the participants with the task (see Online Supplementary Material 1.2 for details). At encoding, participants viewed the events sequentially (5 s each; 0.5-s intertrial interval (ITI)).

The test phase followed the encoding phase and consisted of 35 trials. Fifteen trials were targets – events that contained all three identical elements as studied events. Five trials were scene lures –events that contained identical animals and objects, but the *scenes* were similar exemplars of those seen at encoding. Five trials were *animal* lures –events that contained the identical scenes and objects, but the animals were similar exemplars of those seen at encoding. Five trials were object lures – events that contained identical scenes and animals, but the *objects* were similar exemplars of those seen at encoding. Five trials were foils –events that contained all novel and dissimilar elements from all studied events (see Figure 1B). Participants were instructed to categorize five test event types with five memory judgments (“identical,” “similar scene,” “similar animal,, “similar object,, and “completely new”) by pressing the color-coded keys on the keyboard. We provided a “cheat sheet” with each response and its corresponding color key to ensure that memory failures were not due misremembering response-key mapping. There were no missing responses as the response time was unrestricted. The memory task took approximately 25 min.

##### Pattern completion task

At encoding, participants were told that they would see many different scene-animal-object triads called “events” and that they should pay close attention to all of the different elements including the scene, animal, and object altogether in each event. An example phase preceded the encoding phase in order to acquaint the participants with the task (see Online Supplementary Material 1.2 for details). After the example phase, the encoding phase commenced, in which participants viewed 24 events sequentially (5 s each; 0.5-s ITI).

**Fig. 2:**
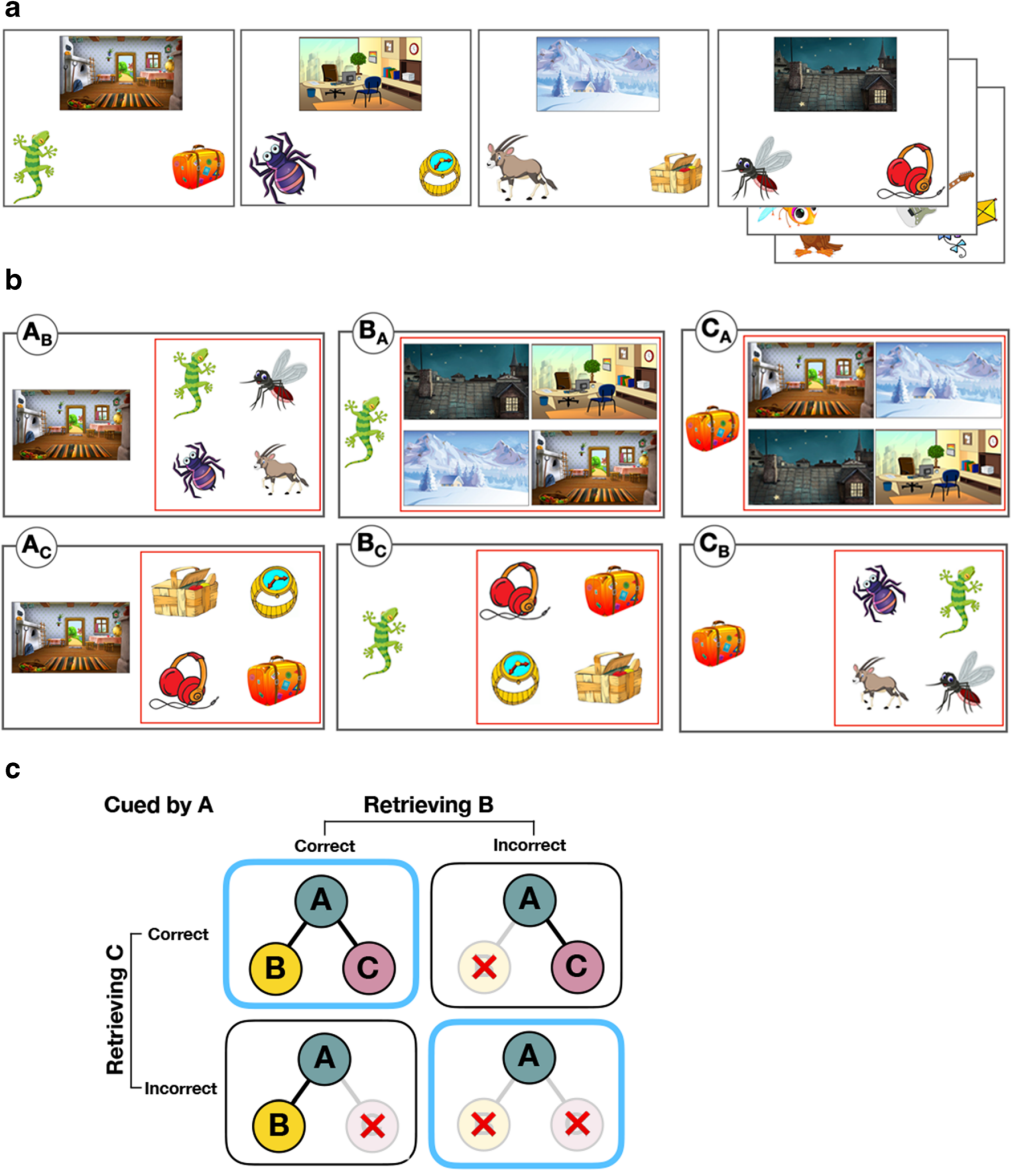
(**A**) Examples of the scene-animal-object. (**B**) Examples of six retrieval types per event in the test phase. Each element of a studied event took turns serving as the cue (item presented on the left side of the screen) and the tested element (one of the four options presented inside the red box). (**C**) A schematic depiction of how the proportion of joint retrieval for A_B_A_C_ pairs was computed for each participant by concatenating the proportion of events in the blue outlined boxes out of the total number of events

Immediately after the encoding phase, participants performed a self-paced four-alternative-forced-choice task that consisted of 144 trials. We tested participants on every possible cue-test combination of each studied event (e.g., cue: scene; test: animal), resulting in six test trials per event, which totaled 144 test trials. On each trial, a cue and four options were presented simultaneously on the screen (see Fig. 2). Among four options, one was a target (the correct item) because it belonged to the same event as the cue. The three lures were same-category elements from different events. The positions of the correct answer were counterbalanced across the entire test phase. Across all 24 events, any given two test trials that had overlapping cue items (e.g., A_B_^1^ and A_C_^1^) or tested items (e.g., B_A_^1^ and C_A_^1^) only shared one foil item (out of three) with respect to their event membership. For example, for the A_B_ test trial of event 1, the foils included the B elements from events 2, 3, and 4, whereas for the A_C_ trial of event 1, the foils included the C elements from events 3, 5, and 7 (one B and one C foil both from event 3). Furthermore, all items served as foils an equal number of times across all 144 test trials. Participants were asked to press the four keys on the number keypad marked with colored stickers that correspond to the four options presented on the right side of the screen. There were no missing responses as the response time was unrestricted. This task took approximately 30 min.

### Verbal intelligence

Prior to the memory tasks, all participants were administered the 45-item American National Adult Reading Test (AMNART; Grober & Sliwinski, 1991) – an American version of the National Adult Reading Test (Nelson, 1982). This test measures the ability to read aloud irregular words. Pronunciation errors were tallied and AMNART-estimated verbal IQ scores were calculated using Grober and Sliwinski’s formula, which accounts for years of education. The AMNART was included as a covariate variable for all statistical analyses.

### Analytical approach

#### Pattern separation

The behavioral expression of pattern separation is characterized by two crucial elements: (1) The detection accuracy and response precision in classify a similar event as similar; and (2) The ability to avoid confusing similar events as old events (reviewed in Yassa & Stark, 2011). To best capture these features of participants’ responses, we computed the confusion matrix for each participant.

##### Confusion matrix

**Fig. 3:**
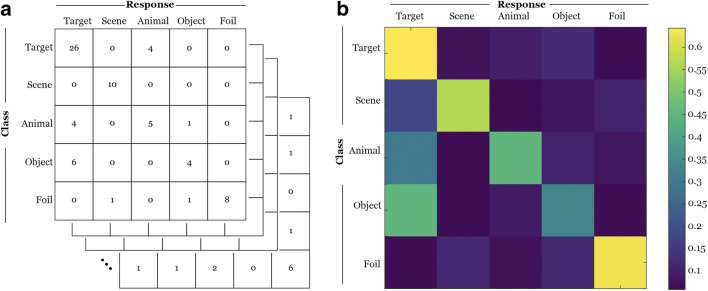
(**A**) A 5 × 5 confusion matrix was constructed for each participant to illustrate the frequency of response (column) to each class of test item (row). (**B**) A group-level confusion matrix with classification accuracy (diagonal cells) and errors (off-diagonal cells). Color intensity illustrates the classification frequency in each cell

**Fig. 4:**
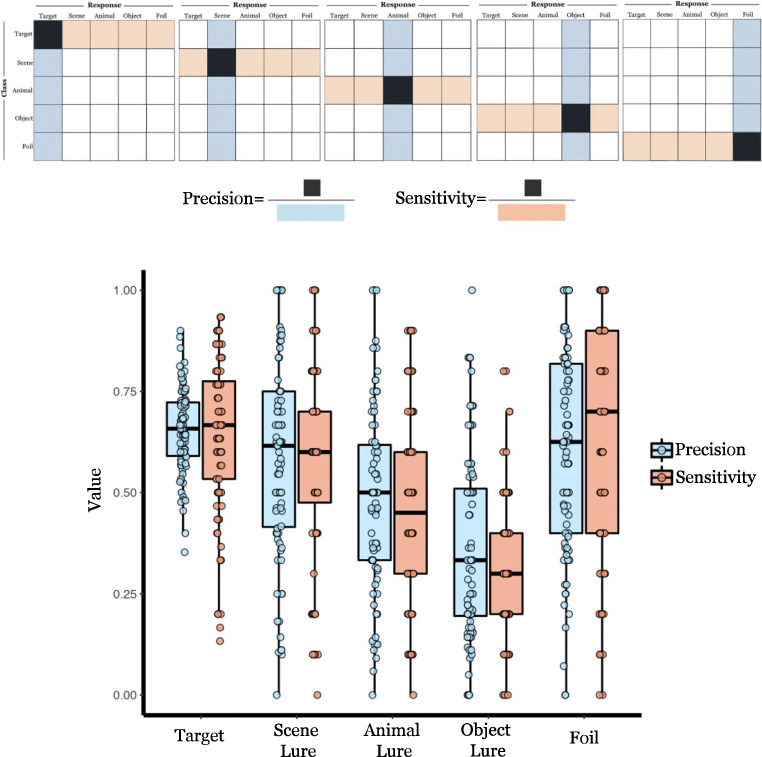
A schematic depiction of the formulas derived from the confusion matrices (**A**) and the distribution of precision and sensitivity for each class of test item (**B**) for the pattern separation task

First, we constructed a 5 × 5 confusion matrix for every participant, with the rows of the matrix representing the classes of the test items (e.g., target, scene lure), and the columns representing a participant’s response frequency. A value in each cell denotes the number of instances in which a participant identified the test items as a given class. The utility of using a confusion matrix is that it effectively handles the multiclass problem. It provides classification accuracy for each class (diagonal cells), and specifies inter-class confusion, i.e., the frequency with which each class is mistaken for each of the other (off-diagonal cells, see Fig. 3). Importantly, from these confusion matrices, we can derive indices of precision and sensitivity for each class (Powers, 2011). Precision of class A is the proportion of correctly identified trials out of the total number of trials a participant labeled the test items as class A (row), therefore capturing the response precision for a given class by a participant. Sensitivity of class A is the proportion of correctly identified trials out of the total number of trials in class A (column), capturing detection performance (see Fig. 4).

Next, we calculated an F1 score combining both precision and sensitivity for each class, which used a harmonic mean of the two indices – a more conservative mean measure as it punishes the extreme values (Powers, 2011). The F1-scores range from 0–1, where 0 indicates no discrimination between a given class from other classes, and 1 indicates a perfect classification for a given class without missing an item from a class or misclassifying items from other classes as a given class.

$$ \mathrm{F}1\ \mathrm{score}\kern0.5em =2\ast \mathrm{Precision}\ast \mathrm{Sensitivity}/\left(\mathrm{Precision}+\mathrm{Sensitivity}\right) $$

Confusing lures as old items is the hallmark of pattern separation failure (reviewed in Yassa & Stark, 2011). Thus, specific kinds of inter-class confusion are crucial to succinctly characterize the underlying processes of pattern separation. In order to approximate the specific ability to pattern separate similar experiences (2), we computed the frequency at which lures were misclassified as targets. To do this, we derived a combined measure by correcting F1 scores for those misclassifications, i.e., we subtracted the frequency with which lures were misclassified as targets from the F1 scores of lures, given that mistaking a similar but different item as an old item is a hallmark of pattern separation failure (reviewed in Yassa & Stark, 2011).

- Scene Lure classification = Scene Lure F1 score – P(“Target ” | Scene Lure)
- Animal Lure classification = Animal Lure F1 score – P(“Target ” | Animal Lure)
- Object Lure classification = Object Lure F1 score – P(“Target ” | Object Lure)

We took a similar approach with targets and foils. For targets, we corrected for the interclass confusion in which that an entirely old event was misclassified as entirely new.^1^ We computed a corrected F1 score for targets by subtracting the frequency in which foils were misclassified as targets from the target F1 score. For foils, we corrected for the interclass confusion in which an entirely new event was mistaken as entirely old. We computed a corrected F1 score for foils by subtracting the frequency with which foils were misclassified as targets from the foil F1 score.

- Target classification = Target F1 score – P(“Foil” | Target)
- Foil classification = Foil F1 score – P(“Target ” | Foil)

#### Pattern completion

##### Estimating retrieval dependency

The retrieval dependency between retrieval successes for different associations within the same event was computed using the same methods as in previous studies (Horner & Burgess, 2013, 2014; Horner et al., 2015; Bisby et al., 2018). Six 2 × 2 contingency tables for the data and the predicted independent model were computed for each participant based on their retrieval accuracy for each pairwise association in order to assess dependency between retrieving two elements when cued by the remaining common element within an event (A_B_A_C_; i.e., cue with A and retrieve B, and cue with A and retrieve C), and the dependency between retrieving a common item when cued by the other two elements within an event (B_A_C_A_; i.e., cue with B and retrieve A, and cue with C and retrieve A). Each 2 × 2 contingency table for the data, for every participant, shows the proportion of events that fall within the four categories: both A_B_ and A_C_ are correct or incorrect, A_B_ correct and A_C_ incorrect, and vice versa. To measure retrieval dependency, we computed the proportion of joint retrieval for the data – defined as the proportion of events in which both associations were either correctly or incorrectly retrieved (cells 1, 1 and 2, 2 of each contingency table; see Fig. 2C). We then averaged this measure across six contingency tables (three tables for the A_B_A_C_ analysis, for each element-type, and three tables for the B_A_C_A_ analysis, for each element-type) for each participant.

The independent model estimates the degree of statistical dependency if retrieval success for different associations within the same event is independent from one another, given a participant’s overall accuracy. If retrievals of event pairs are independent, the probability of the successful retrieval for both A_B_ and A_C_ is equal to P_AB_*P_AC_, where P_AB_ is the probability of retrieving B when cued by A across all events, and similarly for P_AC_ (see Table 1 for full details). The independent model serves as a predicted baseline for which we compare the proportion of joint retrieval in the data. Given that the proportion of joint retrieval in the data scales with accuracy, the main index of retrieval dependency was the difference between the joint retrieval in the data and independent model for each participant – referred to as dependency. If dependency is significantly greater than zero, this provides evidence for significant retrieval dependency (Horner & Burgess, 2014).

**Table 1:** Contingency table for the predicted independent model for proportion of correct and incorrect cued recognition over the total number of events for elements B and C when cued by A

**Cued by A**		**Retrieving B**	
		Correct	Incorrect
**Retrieving C**	Correct	${\Sigma}_{i=1}^N{P}_{AB}{P}_{AC}$	${\Sigma}_{i=1}^N{P}_{AC}\left(1-{P}_{AB}\right)$
	Incorrect	${\Sigma}_{i=1}^N{P}_{AB}\left(1-{P}_{AC}\right)$	${\Sigma}_{i=1}^N\left(1-{P}_{AB}\right)\left(1-{P}_{AC}\right)$

### Statistical analyses

All planned statistical analyses were performed using JASP.

#### Data availability

The stimuli in the memory tasks (https://osf.io/pjy28/) and second-level data (https://osf.io/5k8jx/) are publicly available through the Open Science Framework.

## Results

### Pattern separation task

#### Overall accuracy

Overall accuracy was defined as the proportion of correct responses out of 70 total test trials. On average, young adults’ overall accuracy was 0.56 (*SE* = 0.02), which was significantly above chance (20% given that they were five response choices), *t*(83) = 20.98, *p* < .001, 95% CI for mean difference [0.32, 0.39], *d* = 2.29. There were no sex differences in overall accuracy, *t*(82) = -0.62, *p* = .54, 95% CI for mean difference [-0.09, 0.05], *d* = -0.13, *BF*_01_ = 3.72. Overall accuracy did not significantly differ between participants who completed the pattern separation task first or second, *t*(82) = -0.41, *p* = .69, 95% CI for mean difference [-0.08, 0.05], *d* = -0.09, *BF*_01_ = 4.09. Accuracy on this task and verbal intelligence measured by AMNART showed a significant positive correlation, *r*(82) =0.25, *p* = .02.

**Fig. 5:**
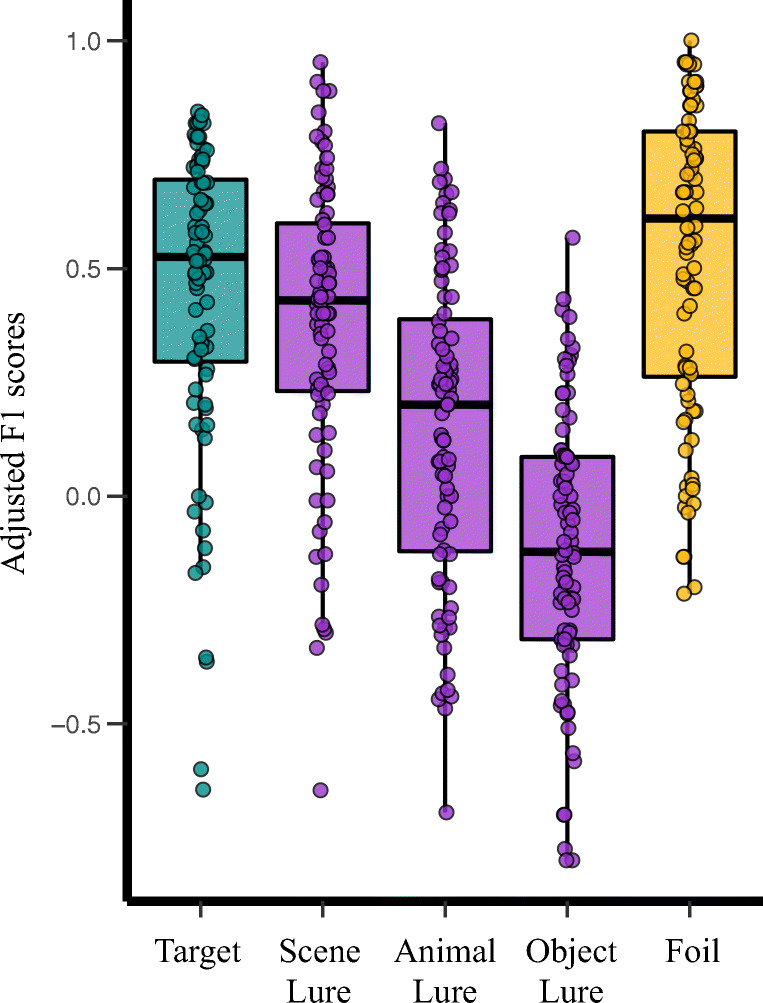
The distribution of adjusted F1 score for each class of test item. The F1 score is the harmonic mean between the sensitivity and precision indices for a given class of test item derived from each participant’s confusion matrix. F1 scores were adjusted for specific misclassification patterns (e.g., misclassify lures as targets, see text)

Frequency of each participant’s response to each class item was calculated in a confusion matrix (see Fig. 3). Precision and sensitivity rates were then calculated for each class of the test item for each participant (see Fig. 4). Finally, we corrected F1 score (harmonic mean of precision and sensitivity) for each class to account for specific inter-class confusion to better characterize discriminability (see Fig. 5).

There was a main effect of test item types, *F*(4, 83) = 75.56, *p* <.001, *partial η*^*2*^ = 0.48. Post hoc comparison with Bonferroni correction revealed that foil (*M* = 0.53, *SE* = 0.04) did not differ from target classification (*M* = 0.45, *SE* = 0.04), *t* = -2.32, *p* = 0.23, 95% CI for mean difference [-0.18, 0.02], *d* = -0.25, but was greater than scene lure (*M* = 0.38, *SE* = 0.04), *t* = -3.76, *p* = .003, 95% CI for mean difference [-0.26, -0.04], *d* = -0.41, animal lure (*M* = 0.15, *SE* = 0.04), *t* = -9.08, *p* < .001, 95% CI for mean difference [-0.50, -0.26], *d* = -0.99, and object lure (*M* = -0.12, *SE* = 0.04), *t* = -13.28, *p* < .001, 95% CI for mean difference [-0.79, -0.51], *d* = -1.45. Target classification did not differ from scene, *t* = 1.79, *p* = 0.77, 95% CI for mean difference [-0.04, 0.18], *d* = 0.20, but was greater than animal, *t* = 6.64, *p* < 0.001, 95% CI for mean difference [0.17, 0.43], *d* = 0.73, and objects, *t* = 12.62, *p* < .001, 95% CI for mean difference [0.44, 0.69], *d* = 1.38.

Lure classification differed across the stimulus categories. Participants were better at classifying scene lures than animal lures, *t* = 5.62, *p* < .001, 95% CI for mean difference [0.11, 0.35], *d* = 0.61, and object lures, *t* = 11.77, *p* < .001, 95% CI for mean difference [0.38, 0.62], *d* = 1.28. Animal lures were classified better than object lures, *t* = 5.49, *p* < .001, 95% CI for mean difference [0.13, 0.40], *d* = 0.60. It is worth noting that the similarity rating is higher for object lure than for scene lure (see Online Supplementary Material 1.1). Thus, differences in lure classification between scenes and objects may be due to the uneven perceptual similarity of the exemplar pairs between these two categories.

### Pattern completion task

#### Overall accuracy

**Fig. 6:**
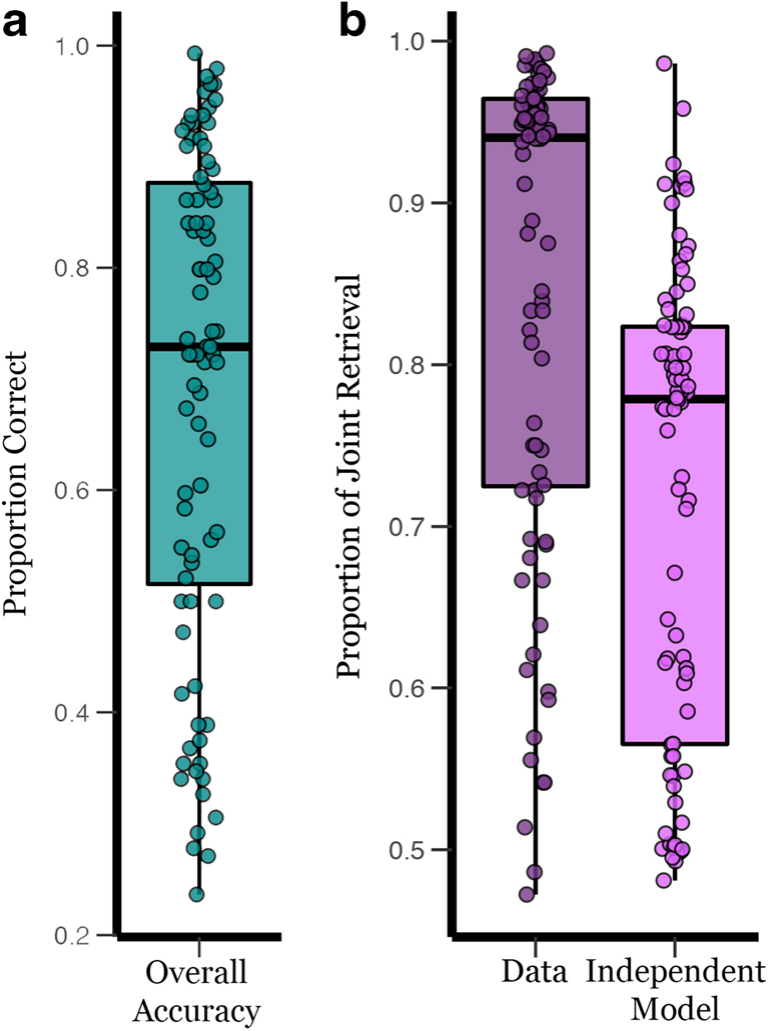
The distribution of the overall accuracy (**A**) and the proportion of joint retrieval of the data and independent model (**B**) of the pattern completion task. Overall accuracy in the pattern completion task is the proportion of correct trials across 144 test trials. The proportion of joint retrieval in the data and the independent model was calculated from the contingency table for each participant

Overall accuracy is defined as the proportion of target selection across 144 test trials. Young adults performed significantly greater than chance level (0.25), *M* = .69, *SE* = 0.02, *t*(83) = 18.21, *p* < .001, 95% CI for mean difference [0.39, 0.49], *d* = 1.99 (see Fig. 6A). There were no sex differences in overall accuracy, *t*(82) = 0.44, *p* = .66, 95% CI for mean difference [-0.08, 0.12], *d* = 0.10, *BF*_01_ = 4.04. Overall accuracy did not significantly differ between participants who completed the pattern completion task first or second, *t*(82) = 1.14, *p* = .26, *d* = 0.25, *BF*_01_ = 2.48. Accuracy and verbal intelligence measured by AMNART showed a trend towards a positive correlation, *r*(82) = 0.18, *p* = .10.

#### Retrieval dependency

To test for evidence of holistic recollection, paired-samples *t*-tests were conducted to examine whether dependency in the data exceeded the independent retrieval model. As expected, we found significant dependency, such that dependency in the data (*M* = 0.85, *SE* = 0.02) exceeded the independent retrieval model (*M* = 0.72, *SE* = 0.02), *t*(83) = 12.35, *p* < .001, 95% CI for mean difference [0.11, 0.15], *d* = 1.35 (see Fig. 6B). These results replicated previous studies showing significant retrieval dependency in young adults using different stimuli (e.g., Bisby et al., 2018; Horner & Burgess, 2013, 2014; Ngo et al., 2019). Together these studies demonstrate that memories for multi-element events may be represented as integrated episodic units.

We also tested whether different stimulus categories yielded uneven retrieval dependency. It has been suggested that scenes may play a robust cuing role in episodic memory (Robin, 2018). Thus, it is possible that scenes may yield stronger dependency compared to other element categories. To test this possibility, we calculated dependency scores for associations that shared the same cue (A_B_A_C_) or tested item (B_A_C_A_) separately for each category. A one-way ANOVA showed a nonsignificant effect of category on dependency, *F*(2, 166) = 1.21, *p* = 0.30, *partial η*^*2*^ = 0.01, suggesting that retrieval dependency did not differ among the three categories of elements.

### Pattern completion and pattern separation correlations

#### Dependency and lure classification correlations

**Fig. 7:**
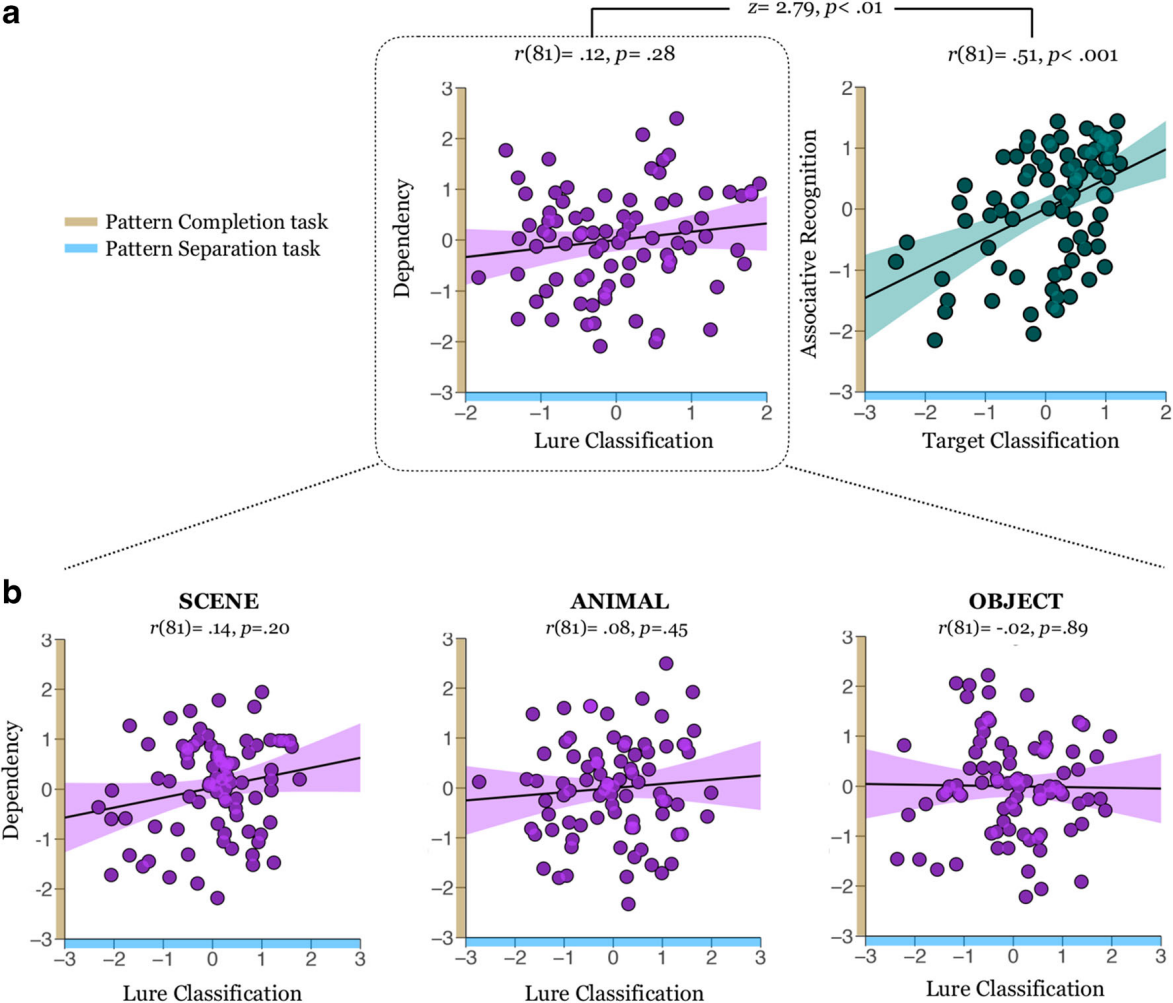
Scatterplots of the standardized residuals illustrating the relation between dependency on the pattern completion task and lure classification on pattern separation task (**A, left**), between associative memory on the pattern completion task and the target classification on the pattern separation task (**A, right**), and between dependency and lure classification for each stimulus category separately (**B**). All scatterplots depict the partial correlations after partialing out AMNART score

To probe the behavioral relation between pattern separation and completion, we tested whether individual differences in lure classification and dependency co-varied with one another. We averaged the adjusted F1 scores from the three lure categories to yield one overall lure classification index. Dependency was a difference between the proportion of joint retrieval in the data and the independent model of retrieval. A partial correlation between lure classification and dependency, with AMNART as a covariate, revealed a nonsignificant correlation, *r*(81) = .12, *p* = .28, 95% CI [-0.09, 0.32], *BF*01 = 4.05 (see Fig. 7A).

We further tested the possibility that pattern separation and completion only show related performances at the category-specific level, we correlated lure classification and dependency within each category separately (scene, animal, object; see pre-registration). Lure classification and category-specific dependency did not correlate for scene, *r(*81) = .14, *p* = 0.20, 95% CI [-0.07, 0.35], *BF*01 = 3.22, animal *r(*81) = .08, *p* = 0.45, 95% CI [-0.13, 0.29], *BF*01 = 5.56, or object, *r(*81) = -0.02, *p* = .89, 95% CI [-0.23, 0.20], *BF*01 = 7.27, after partialing out AMNART (see Fig. 7B).

These results suggest that lure classification and holistic recollection may be behaviorally separable processes such that individual differences in one facet of episodic capacity do not track those in the other.

#### Pairwise associative memory and target classification correlation

Next, we reasoned that indices of general episodic memory performance – nonspecific to retrieval dependency or lure classification – should correlate between the two tasks as they both tap general episodic capacity. For the pattern separation task, lure classification specifically indexes pattern separation, whereas *target classification* indexes the abilities to recognize old events. For the pattern completion task, dependency indexes holistic recollection, whereas *overall accuracy* indexes pairwise associative recognition, irrespective of event membership. Both of these processes are general memory performances that are not uniquely attributed to pattern separation or pattern completion. Therefore, we predicted that overall accuracy in the pattern completion task and target classification in the pattern separation task would relate to one another. We treated this analysis as a “control” correlation analysis to ensure that the nonsignificant correlation between lure classification and retrieval dependency was not due to potential superficial differences between the two tasks, or due to power issues (see pre-registration). Indeed, they were strongly correlated, *r*(81) = .51, *p* < .001, after controlling for AMNART (see Fig. 7A). Crucially, this correlation significantly exceeded the dependency versus lure classification correlation, *Z* = 2.79, *p* < 0.01.

In our pre-registration, we planned to assess pattern separation performance using the raw lure identification rate (i.e., identifying a test event that contained a similar scene to a scene lure trial). However, raw lure identification does not adjust either for response bias or for the specific false alarm rate relevant to pattern separation (i.e., misclassifying a lure as a target). Thus, we employed a confusion matrix approach to estimate pattern separation. The correlational results using the pre-registered approach are reported in Online Supplemental Material 2.2.

## General discussion

The hippocampus supports episodic memory capacity by (1) preserving distinctive features of specific past experiences, and (2) by recapitulating constituent parts of an episodic memory altogether at retrieval. Pattern separation and completion are the underlying neurocomputationally distinct processes that work in concert to support different aspects of episodic memory capacity. This study tested whether individual differences in the behavioral indices of these processes would relate to each other using an individual differences approach. In a novel combination of behavioral tasks of pattern separation and completion, we show that retrieval dependency and lure classification indeed do not track each other. These findings demonstrate a degree of inter-independence of the two processes, suggesting that different hippocampal computations play complementary roles in supporting different facets of episodic memory. The nonsignificant correlation between holistic recollection and lure classification aligned with our predictions, and was supported by an analysis using Bayes factors, which suggests that the null hypothesis is four times more likely than the alternative hypothesis. Further, gross performances on the two tasks – nonspecific to pattern separation and completion – were significantly correlated. Importantly, the gross-performances correlation was greater than that of the dependency versus lure classification correlation. These results suggest that certain aspects of the two tasks tap general episodic capacity, and that our null finding was unlikely to be due to power issues.

Our findings shed light on the debate on the computational interplay between pattern separation and pattern completion (for a discussion, see Hunsaker & Kesner, 2013). The behavioral dissociation between lure discrimination and retrieving old events from partial cues aligns with neuropsychological findings in a patient with selective dentate gyrus damage (Baker et al., 2016). As expected, this patient was severely impaired at lure discrimination on the MST. In addition to the MST, the authors administered the Memory Image Completion (MIC) task, which evaluates recognition memory for scenes (old and new) presented at varying degrees of completeness during test. Interestingly, the patient was spared on recognizing old scenes at all levels of completeness, suggesting that abilities to complete a true old memory from partial cues was neither impaired or enhanced compared to healthy controls. However, he was impaired in correctly rejecting novel scenes at all levels of completeness compared to healthy controls, suggesting an increased tendency to pattern complete in the presence of noisy cues. Based on these findings, DG damage appears to be linked to the selective deficits in lure discrimination (i.e., poor lure rejection in both the MST and MIC), while sparing the ability to reconstruct old experience based on partial cues (i.e., intact target recognition in the MIC). This neuropsychological evidence that an impairment in lure discrimination does not necessarily come with an impairment (or increase) in pattern completing an old event converges with our current findings in healthy adults.

Our study employed separate paradigms from the pattern separation and completion literature that provide independent behavioral indices. We implemented critical methodological and analytical modifications to specifically probe their behavioral relation. Methodologically, we equated the encoding format of both tasks using multi-element events, departing from other MST-task variants, which typically test individual items (e.g., as reviewed in Yassa & Stark, 2011). This design allowed us to examine the pattern separation-pattern completion relations at the category-agnostic and category-specific levels. Analytically, we leveraged computational principles from confusion matrices to extract one index of lure classification, accounting for hit rate, response precision, and false alarm.

It is important to note that there is variation in the behavioral paradigms designed to approximate pattern completion. Based on the definition of pattern completion as recovering an old experience from partial or degraded cues, some tasks have operationalized partial cues as fragments of learned scenes (Vieweg et al., 2015), whereas in the multi-element event task, partial cues are defined as elements within complex events (Horner & Burgess, 2013, 2014). Others have operationalized degraded cues as noisy cues through the use of similar lures that include both the original input and added novel features (e.g., perceptually similar object exemplars) (Yassa & Stark, 2011). The nature of the cues presented at test may impact the degree to which pattern completion is engaged (O’Reilly & McClelland, 1994). Importantly, it has been suggested that the use of partial cues as opposed to noisy cues in behavioral paradigm may preferentially engage pattern completion (reviewed in Liu, Gould, Coulson, Ward, & Howard, 2016).

Further, when assessing pattern completion behaviorally, it may be important to consider the difference between a gravitation towards pattern completion in the presence of noisy cues and the capacity to pattern complete a true old event based on partial cues. Models of aging propose that over-activity of the CA3 auto-associative function leads to the overexpression of old information at the expense of discriminating new information (Wilson, Gallagher, Eichenbaum, & Tanila, 2006). Relative to young adults, older adults show a greater tendency to falsely recognizing lures as old scenes (Stark, Yassa, Lacy, & Stark, 2013; Vieweg et al., 2015; Yassa and Stark, 2011). At the same time, older adults are also less likely to pattern complete target items as old based on partial inputs (Vieweg et al., 2015). A reduction in the abilities to recover the learned experiences from partial cues may co-occur with an increase in a bias towards pattern completion old events when in the presence of noisy cues. However, the inferences of pattern separation and completion based on one behavioral continuum of lure discrimination performance may not make this distinction obvious.

Our work presented the initial evidence that capacities of remembering a past event in its totality and remembering individual elements with high resolution do not scale with one another in healthy young adults. These processes likely play complementary roles and contribute to distinct properties of complex episodic capacities. Interpretation of pattern completion using behavioral measures would also benefit from a systematic examination of whether tasks designed to assess pattern completion correlate with one another and how each may relate to pattern separation. Future research should tackle a full treatment of pattern separation and pattern completion dissociation by combining behavioral paradigms and neuroimaging methods.

### Author Note

We would like to thank Ying Lin, Elizabeth Eberts, Lizi Zhong, Richard Ho, Rebecca Adler, Emily Cerimele, and Nadhia Engle, for their help with stimuli development and/or with data collection. This work was supported in part by National Institutes of Health grants F31HD090872 to C.T.N, RO1 MH091113 to I.R.O., and R21 HD098509 to I.R.O. and N.S.N. The content is solely the responsibility of the authors and does not necessarily represent the official views of the National Institute of Health. The authors declare no competing or conflicting financial interests.

## Electronic supplementary material

ESM 1 (CSV 1 kb)

## Footnotes

1 The same results on the correlation between the two tasks were found when target classification was uncorrected.
